# Microglial Galectin3 enhances endothelial metabolism and promotes pathological angiogenesis via Notch inhibition by competitively binding to Jag1

**DOI:** 10.1038/s41419-023-05897-8

**Published:** 2023-06-28

**Authors:** Zi-Yi Zhou, Tian-Fang Chang, Zhi-Bin Lin, Yu-Tong Jing, Li-Shi Wen, Ya-Li Niu, Qian Bai, Chang-Mei Guo, Jia-Xing Sun, Yu-Sheng Wang, Guo-Rui Dou

**Affiliations:** 1 Department of Ophthalmology, Eye Institute of Chinese PLA, Xijing Hospital, Fourth Military Medical University, Xi’an, 710032 China; 2 Department of Hepatobiliary Surgery, Xijing Hospital, Fourth Military Medical University, Xi’an, 710032 China

**Keywords:** Mechanisms of disease, Peripheral vascular disease

## Abstract

Microglia were considered as immune cells in inflammation until their angiogenic role was widely understood. Although the pro-inflammatory role of microglia in retinal angiogenesis has been explored, little is known about its role in pro-angiogenesis and the microglia–endothelia interaction. Here, we report that galectin-3 (Gal3) released by activated microglia functions as a communicator between microglia and endothelia and competitively binds to Jag1, thus inhibiting the Notch signaling pathway and enhancing endothelial angiogenic metabolism to promote angiogenesis. These results suggest that Gal3 may be a novel and effective target in the treatment of retinal angiogenesis.

## Introduction

Pathological angiogenesis is a major cause of irreversible blindness in ocular diseases, including diabetic retinopathy (DR), age-related macular degeneration (AMD), retinal vein occlusion, and retinopathy of prematurity (ROP) [1]. These conditions feature increased endothelial sprouting and proliferation with consequent excessive neo-vasculature, and disturbance of endothelial homeostasis is the principal trigger for these cellular events [2, 3]. Our team and others have previously demonstrated that Notch signaling is critical to maintain endothelial quiescence [4–7]. Notch activation in mice has also been reported to reduce pathological angiogenesis in models of ROP and AMD, by promoting endothelial quiescence and regulating endothelial metabolism [8–10]. However, endothelial Notch activity could be indirectly regulated by adjacent cells, such as macrophages/microglia in the angiogenic vascular niche, and by factors that mediate angiogenic cross-talk between endothelial cells (ECs) and their neighboring cells [11, 12]. Thus, understanding cellular communication and the mechanisms underlying the angiogenic niche may lead to efficient therapeutic strategies for pathologic retinal angiogenesis.

In the retinal microenvironment, microglia, a specialized type of immune cell resident in the central nervous system and retina, constantly monitor slight subtle changes in their surroundings and maintain balanced functions by communicating with other retinal cells [13–17]. Microglia reportedly play a pivotal role in shaping developmental retinal vascular formation and are responsible for pathological angiogenesis upon insults [13, 15]. During angiogenesis, they gather around neovascular tufts and interact with endothelial apical cells to promote vascular sprouting and pruning [18–20]. More recently, evidence increasingly points towards the paracrine effect of microglia to regulate other nearby cells [21–23]. miRNA in extracellular vesicles (EVs) may provide a bridge between microglia and adjacent cells [24]. Bi et al. [25] found that resident microglia regulate pre-sympathetic neurons to maintain the balance of sympathetic outflow by releasing platelet-derived growth factor B (PDGFB), providing an example of complex interactions between microglia and adjacent cells in normal and pathological conditions.

As the only known galectin member of the chimera-type family comprising a C-terminal domain (CRD) and N-terminal non-CRD for carbohydrate binding, galectin-3 (Gal3) has been found in the inflammatory response, and its expression is increased in the activated microglia/macrophage upon several stimuli including ischemic injury [26, 27]. Gal3 can be expressed in the cytoplasm, nucleus, and membranes [28, 29] and released into the extracellular space [30, 31]. The different subcellular localizations of Gal3 together with its possible posttranslational modifications are likely to affect its function. Despite its known role in inflammatory regulation, Gal3 may also be involved in neovascular diseases [32]. Gal3 knockdown greatly reduced vascular endothelial growth factor (VEGF)-and basic fibroblast growth factor (bFGF)-mediated angiogenesis [31]. Cano et al. [33] found that Gal3 promoted the activation of the VEGF/VEGFR2 signaling pathway. However, the direct regulation of Gal3 on ECs remains to be fully elucidated.

Several novel molecular pathways for Gal3 were recently uncovered. It is a natural ligand for triggering receptors expressed on myeloid cells (TREM2), Toll-like receptor 4 (TLR4), and insulin receptor (IR) [34]. Gal3 regulates glycosylation receptors by binding to and modifying the glycosylation sites of extracellular membrane glycoproteins [35, 36]. The ligand/receptor of Notch has a variety of glycosylation modifications such as *O*-glucan, *O*-fucoidan, and *N*-glycan [37, 38], providing a glycosylation site for Gal3, thereby influencing activation or inhibition of the Notch pathway. It is therefore feasible that Gal3 might bind to Notch ligands on the cell membrane surface and nearby regulated cells. In the current study, we established an experimental retinal neovascular (NV) mouse model and performed in vitro cell studies to explore the role of microglial Gal3 and its possible role in Notch signaling.

## Results

### Gal3 was upregulated and mainly expressed in activated microglia in retinal angiogenesis

**Fig. 1:**
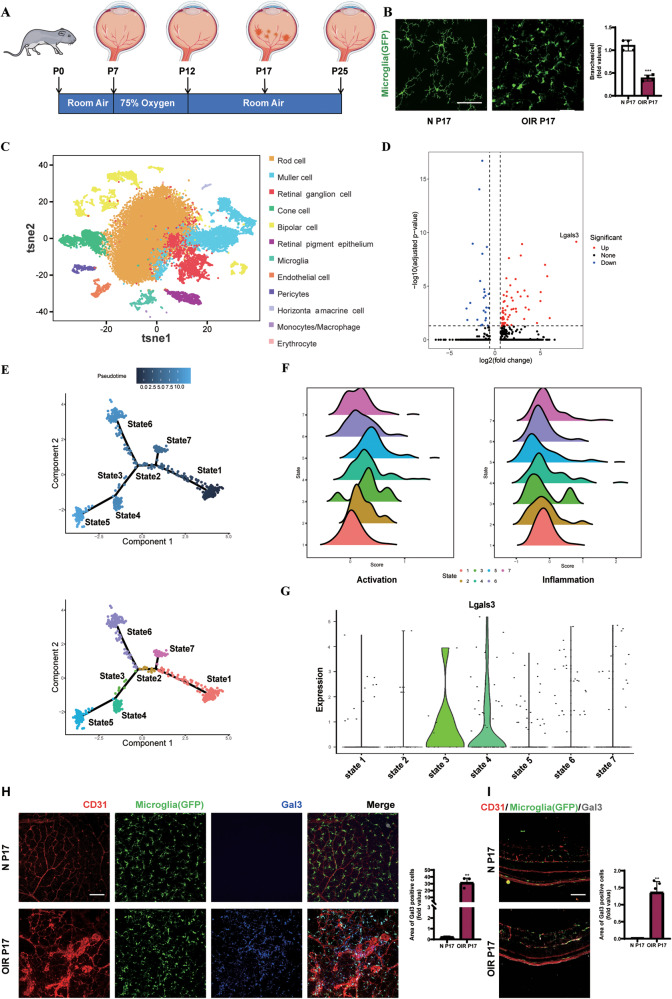
Microglia activated and upregulated the expression of Gal3 in the OIR retina. **A** The schematic of the OIR model in mice. Exposure to hyperoxic (75% O_2_) in mice from P7 to P12 induced the dropout of retinal vascularization. Ischemia insult triggered pathological neovascularization (NV) and peaked on P17. The NV regressed from P17 to P25. **B** Retina samples were collected from the normal and OIR groups of CX3CR1-GFP mice on P17. The expression of CX3CR1 (Green, GFP) on microglia was analyzed by IF staining. Scale bar, 100 µm (*n* = 4 per group). **C** The total retinal cells of the control and OIR group were analyzed by single-cell RNA-seq. **D** The top 20 genes upregulated and top ten genes downregulated in microglia of the OIR group were analyzed by R. Lgals3 was the most significant gene upgraded then. The trajectory analysis in the pseudotiome of microglia were presented in (**E**), showing the differentiation process by gradient colors and states in differentiation by different colors. **F** Ridge plot showed the activation and inflammation level of each state in trajectory analysis according to a set of genes. The expression of lgals3 in all the seven states of microglia was shown in (**G**). **H**, **I** IF staining of retinal vessels (CD31, red), microglia (CX3CR1, GFP), and Gal3 (blue) on P17 in the flat-mounted cross-section from normal and OIR groups. Scale bar, 100 µm (*n* = 3 per group). Bars = means ± SD; **P < 0.01; ***P < 0.001.

To establish a mouse model of retinal angiogenesis by oxygen-induced retinopathy (OIR), mouse pups of CX3CR1-GFP mice were exposed to hyperoxia (75% oxygen) from postnatal day 7 (P7) to P12 to induce vaso-obliteration, and then returned to room air. The maximal preretinal NV occurred at P17, followed by a phase of vascular regression to P25 [39] (Fig. 1A). By comparisons between groups, we found that the morphology and distribution of microglia expressing GFP changed significantly (Fig. 1B). Microglia in the control group showed a branched state, with small cell body and long process on P17, and were evenly distributed in the retina without apparent aggregation. However, a series of microglial changes appeared in the OIR group. The cell body enlarged significantly and the process shortened. Moreover, microglia were distributed unevenly, mainly gathered in and around the neovascularization cluster (Fig. S1A). These results show that microglia were significantly activated in the OIR model. To further explore the changes in microglia after ischemic insult and considering the small portion of microglia in the retina, single-cell RNA sequencing (scRNA-seq) was performed. We sequenced the whole retinal cells in the OIR model and the control group, subdivided the cell subsets according to the retinal cell-specific markers from a previous publication [40, 41], and divided all the retinal cells into 12 clusters (Fig. 1C). One of the most apparent differences between microglia in normal and OIR groups was the upregulation of lgals3 (Fig. 1D). To divide the microglia into subclusters, we performed a pseudotime trajectory analysis in both the OIR and control groups. Noise from myeloid cells was eliminated by marking retinal microglia by the positive expression of the fractalkine receptor CX3CR1 and the negative expression of C-C motif chemokine receptor-2 (CX3CR1 + CCR2- microglia) [42–44] and the microglia were divided into seven states according to their differentiation trajectories (Fig. 1E). The activation and inflammation level of the microglia in these seven states was evaluated in the ridge plots (Fig. 1F). Furthermore, states 3 and 4 with a relatively high level of activation and inflammation showed a prominently high level of lgals3 expression (Fig. 1G) as well as other activation-related genes (Fig. S1B), revealing that lgals3 mainly expressed by activated microglia in the OIR retina.

We then explored the distribution of Gal3 (encoded by Lgals3) via immunofluorescence (IF) staining in the retinas of CX3CR1-GFP mice. In the OIR retina, Gal3 (encoded by lgals3) expression was highly upregulated and co-located with microglia (Fig. 1H, I) and was significantly increased in either protein or mRNA level in the OIR group (Fig. S1C, D). To further determine the source of Gal3, we performed fluorescence in situ hybridization (FISH) in the CX3CR1-GFP mice and co-stained microglia with IBA1 in the wild-type (WT) mice on frozen retinal sections. The results indicated that Gal3 was similarly expressed by microglia (Fig. S1E)

### Microglia-derived Gal3 played a pivotal role in retinal angiogenesis

**Fig. 2:**
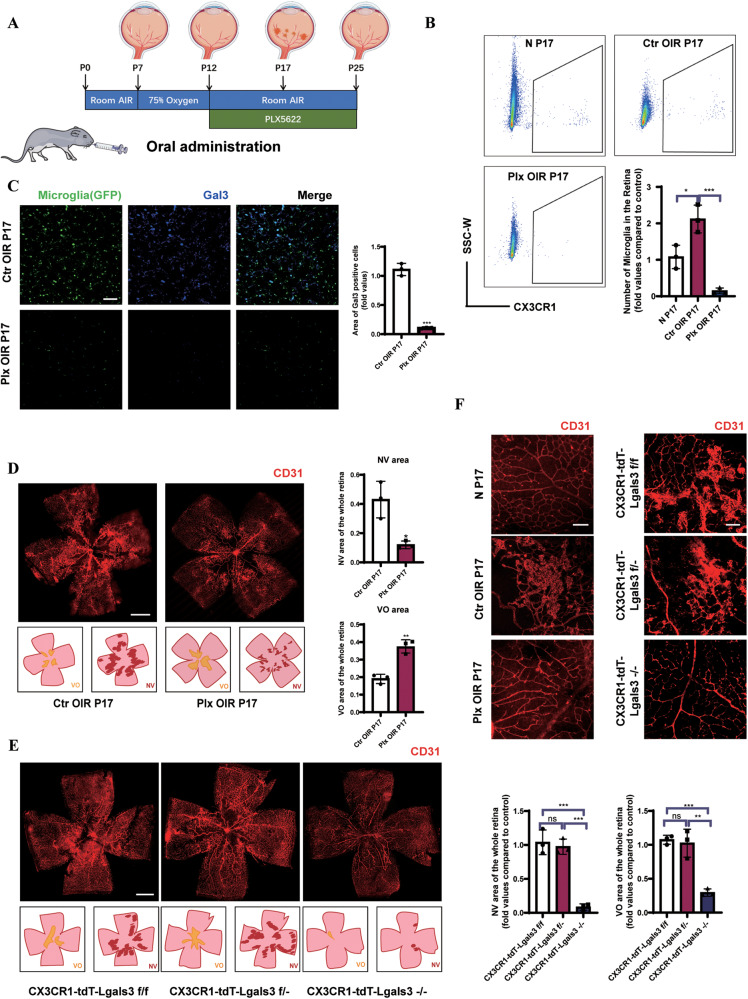
Removal of microglia and conditional knockout of Gal3 in microglia alleviate retinal angiogenesis. **A** The schematic of PLX5622 administration in the OIR mice by gavage from P12 since removed to normoxic condition. **B** The number of CX3CR1+ microglia were evaluated by FACS and quantified in the normal, OIR, and PLX group (*n* = 3 per group). **C** IF staining of microglia (CX3CR1, GFP) and Gal3 (blue) in the flat-mounted retinal tissue from the Ctr OIR group and Plx OIR group showed the removal of microglia in the retina by the administration of Plx5622. (*n* = 3 per group) Scale bar, 100 µm. **D** IF staining was used to evaluate vessel clusters (CD31, red) and avascular areas. (*n* = 3 per group). Scale bar, 500 µm. **E** The whole retinas of CX3CR1-tdT-Lgals3(f/f, f/−. −/−) mice were collected and the vessels were stained with CD31 (red). (*n* = 3 per group). Scale bar, 500 µm. The specific morphology of blood vessels was captured in (**F**). (*n* = 3 per group). Scale bar, 100 µm. Bars = means ± SD; *P < 0.05; **P < 0.01; ***P < 0.001, NS no significance.

Then we removed microglia in the mouse retina using a colony-stimulating factor 1 receptor (CSF1R) inhibitor PLX5622 by gavage of the OIR mice from P12 to P17 (Fig. 2A). On P17, flow cytometry and IF staining were used to confirm the removal efficiency. The number of microglia increased significantly in the OIR group but they almost disappeared after PLX5622 administration (Fig. 2B and S2A). After microglia removal, Gal3 expression reached a minimum (consistent with it being derived from microglia) (Fig. 2C and S2B, C), and neovascular clusters in the angiogenic stage were clearly reduced (Fig. 2D).

Because Gal3 was significantly upregulated in activated microglial cells, we explored the role of the Gal3 protein in the pathological process. In tamoxifen-induced microglial conditional Gal3 knockout mice (CX3CR1-tdT-Lgals3−/−) (Fig. S3A–C), less neovascular area (NV area) and less vaso-obliterated area (VO area) were observed (Fig. 2E), demonstrating the angiogenic role of Gal3 in the retina. Few changes in the number of microglia in the retina was observed after Gal3 knockout (Fig. S2D). Diminished pathological vessel clusters and normalized microvessels were found in both the microglia-removed retina and conditional microglia-Gal3 knockout retina (Fig. 2F), suggesting a pivotal role of microglial Gal3 in retinal angiogenesis.

### Microglia-derived Gal3 promoted pathological angiogenesis by directly regulating ECs

To further explore the role of Gal3 in retinal angiogenesis, we activated microglia by hypoxia in vitro to simulate pathology. In hypoxia, the morphology of microglia changed (Fig. S4A, B), with larger cell bodies and shorter processes. The expression of Gal3 by microglia was also upregulated after 4 h of hypoxia and peaked at 8 h (Fig. S4C). The expression of inflammatory factors such as interleukin (IL)-1β and IL-6, was also tested after hypoxia (Fig. S4D) to gauge microglial activation.

**Fig. 3:**
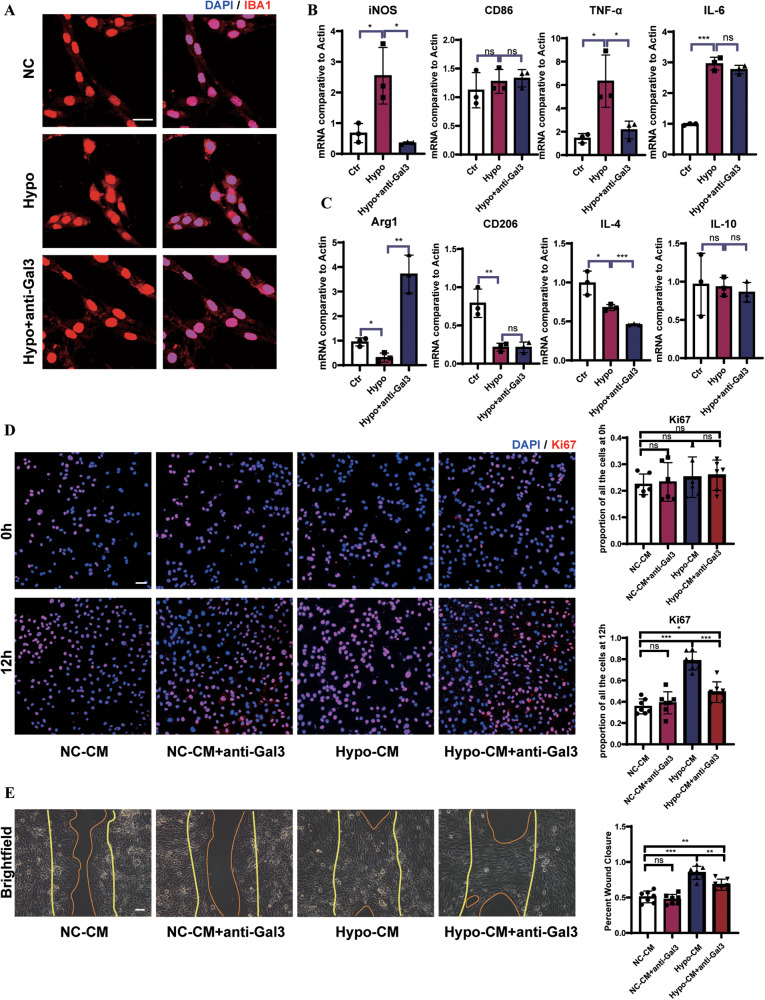
Gal3 regulated mainly on ECs rather than on microglia. BV2 microglia were cultured in hypoxia for 12 h, followed by immunostaining for IBA1(**A**). Scale bar, 100 µm. **B**, **C** mRNA expression of M1-related genes (iNOS, CD86, TNF-α, and IL-6) and M2-related genes (Arg1, CD206, IL-4, and IL-10) were determined by qRT-PCR (*n* = 3 per group). **D** HUVECs cultured by conditioned media collected from BV2 microglia in normoxia and hypoxia for 12 h, followed by immunostaining for Ki67 (red). (*n* = 7 per group). Scale bar, 100 µm. **E** HUVECs were wounded with a p20 pipette tip and then cultured by conditioned media from BV2 microglia in normoxia and hypoxia for 12 h. Photographs were taken immediately and after 8 h (*n* = 7 per group for wound assay and two fields were calculated per sample). Scale bar, 100 µm.Bars = means ± SD; *P < 0.05; **P < 0.01; ***P < 0.001, NS no significance.

Gal3 may have both pro-and anti-inflammatory effects directly on macrophages [45, 46]. The M2 subtype of microglia functions as a pro-angiogenic factor [47, 48]. Here we observed that anti-Gal3 treatment-induced changes in the morphology of BV2 cells, indicating that neutralization of Gal3 reversed the activated state of microglia in hypoxia (Fig. 3A). Further, the expression of classic M1 and M2 markers and several cytokines were tested. We found that the neutralization of Gal3 in activated microglia inhibited rare cytokines of M1 and M2 subtypes (Fig. 3B, C), suggesting that Gal3 may have a different mechanism.

Intriguingly, the proliferation of murine brain microvascular endothelial cell line bEnd.3 cells cultured in the conditioned media from the activated microglia induced by hypoxia were enhanced compared with controls (Fig. 3D) and their capacity for cell migration was promoted, a change which may contribute to enhanced wound healing (Fig. 3E). This effect by conditioned media from activated microglia was dampened once the anti-Gal3 was added to the media (Fig. 3D, E). Gal3 in the cell culture supernatant was detected, implying that Gal3 secreted by microglia could be stimulated by hypoxia (Fig. S5A). Similarly, when recombinant human Gal3 (rhGal3) was adopted to treat human umbilical vein ECs (HUVECs) and found that cell proliferation and migration was promoted compared with the control group (Fig. S5B, C). Taken together, the above observations suggest that Gal3, as a microglial paracrine factor, promoted pathological angiogenesis by directly regulating ECs.

### Microglial Gal3 disrupted endothelial homeostasis and regulated endothelial metabolism

**Fig. 4:**
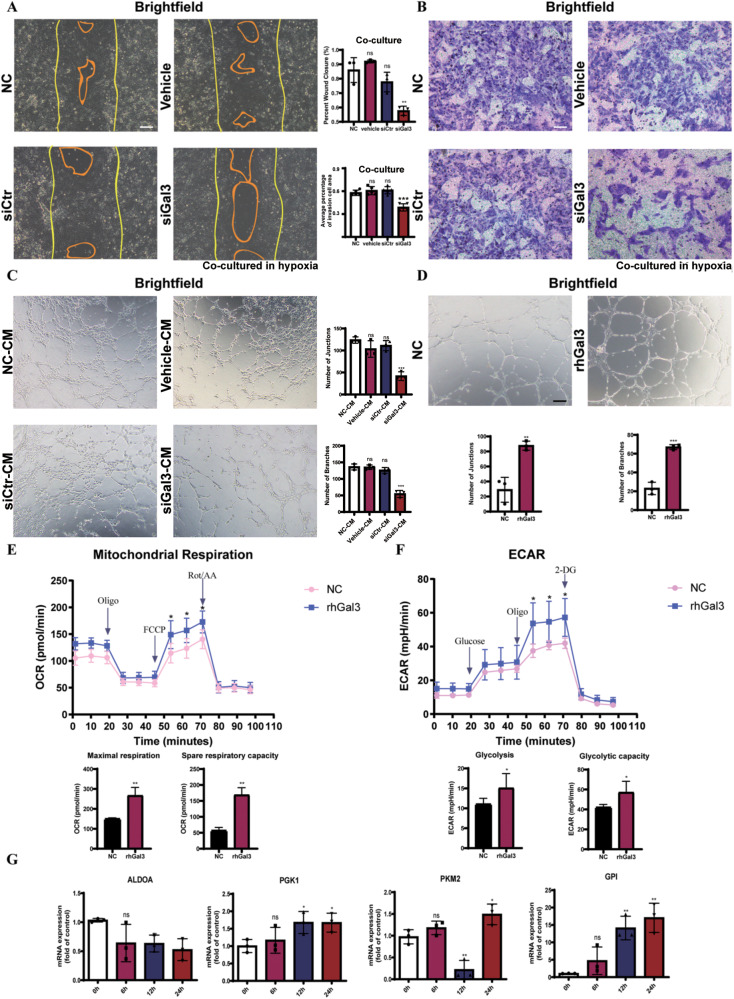
Gal3 promoted endothelial proliferation and lifted endothelial metabolism. **A** The bEnd.3 cells and were wounded with a p20 pipette tip and then were cocultured with BV2 transfected with siGal3 as compared to those with BV2 transfected with siRNA control and vehicle (lipo2000). Photographs were taken immediately and after 8 h (*n* = 3 per group for wound assay and two fields were calculated per sample). Scale bar, 100 µm. **B** The capacity of cell migration of bEnd.3 cells cocultured with microglia after 12 h in hypoxia was evaluated by transwell assay (*n* = 4 per group). Scale bar, 100 µm. **C** Lumen formation of bEnd.3 cells cultured by conditioned media was detected and compared between the groups after 6 h. (*n* = 3 per group). Scale bar, 100 µm. **D** The capacity of lumen formation of HUVECs cultured by normal ECM and ECM with rhGal3 after 6 h of stimulation (*n* = 3 per group). OCR (**E**) and ECAR (**F**) measured with the Seahorse XFe24 analyser in HUVECs cultured by normal ECM and ECM with Gal3 for 12 h (*n* = 5 per group). **G** mRNA level of enzymes in glycolysis was tested in HUVECs cultured by normal ECM and ECM with rhGal3 after 12 h by qRT-PCR, including ALDOA, PGK1, PKM2, and GPI. (*n* = 3 per group). Bars = means ± SD; *P < 0.05; **P < 0.01; ***P < 0.001, NS no significance.

To explore the function of Gal3 in endothelia-microglia interaction and EC regulation, we knocked down the expression of Gal3 in BV2 microglia cells (Fig. S5D, E). The endothelial bEnd.3 cells were then cocultured with the BV2 cells in hypoxia. The capacities of bEnd.3 cells for cell migration (Fig. 4A) and cell invasion (Fig. 4B) were significantly impaired when cocultured with the BV2 cells in which the expression of Gal3 was knocked down.

We then cultured bEnd.3 cells with conditioned media from BV2 cells. Cell proliferation capacity was tested using a cell counting kit-8 (CCK8) assay, which showed the proliferation activity of bEnd.3 cells was reduced with Gal3 knockdown (Fig. S5F). The lumen formation capacity of bEnd.3 cells was also reduced by cultured with conditioned media from Gal3 knockdown group (Fig. 4C). The results indicate that microglia-derived Gal3 regulated microglia–endothelia interaction and promoted endothelial proliferation in vitro.

To explore how Gal3 regulated ECs, we added exogenous rhGal3 to HUVECs and found that the lumen formation capacity of HUVECs was enhanced (Fig. 4D). We measured oxygen consumption rate (OCR) as an indicator of the level of metabolism in HUVECs using Seahorse XF. OCR could reflect mitochondrial respiration and extracellular acidification rate (ECAR) could in turn reflect glycolysis flux. The results indicated that the metabolic level of HUVECs was raised after stimulation by Gal3 (Fig. 4E, F). Using quantitative real-time PCR, we focused on the mRNA level of the genes of glycolytic metabolism in HUVECs. Consistent with the results in ECAR, most genes encoding rate-controlling enzymes in glycolysis were upregulated after being cultured with rhGal3 (Fig. 4G). The changes in endothelial metabolism explained the proliferation and activation of ECs in microglia–endothelia interaction.

### Gal3 regulated angiogenesis by binding to Jag1 and blocking the Notch signaling pathway

**Fig. 5:**
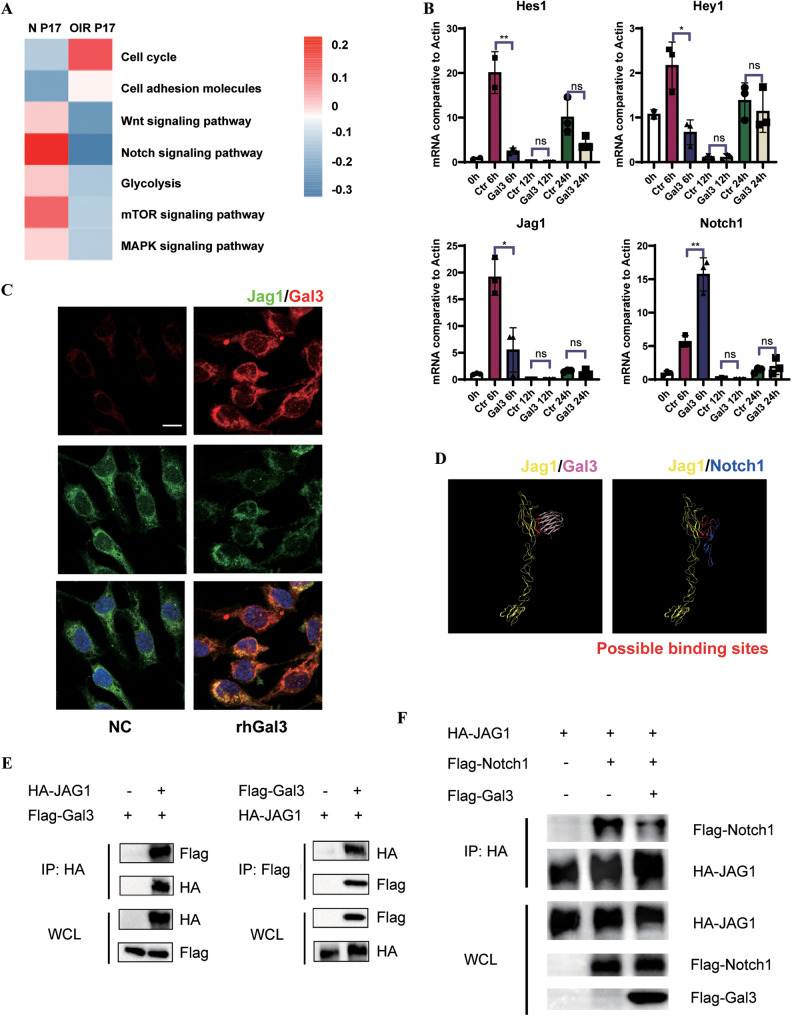
Gal3 regulated ECs via blocking the Notch pathway by competitively binding to Jag1. **A** GSVA scores on gene sets related to cellular proliferation and metabolism comparing transcripts from scRNA-seq of P17 normal and OIR retinas were analyzed (**B**) mRNA expression of Hes1, Hey1, Jag1, and Notch1 were determined in HUVECs cultured by normal ECM and ECM with rhGal3 immediately and after 6, 12, and 24 h by qRT-PCR (*n* = 3 per group). **C** IF staining of Jag1 (green), Gal3 (red), and DAPI (blue) in HUVECs cultured by normal ECM and ECM with rhGal3 for 6 h. (*n* = 3 per group). Scale bar, 100 µm. **D** Molecular docking prediction model of Jag1 (yellow) with Gal3 (pink) and Jag1(yellow) with Notch1(blue). The possible binding sites were marked with red. **E**, **F** IP experiments were conducted to validate the competitive binding of Gal3 to Jag1 with Notch1. (*n* = 3 per group). Bars = means ± SD; *P < 0.05; **P < 0.01, NS no significance.

We explored the downstream mechanisms in the regulatory effect of Gal3 on ECs. Analysis of the enrichment pathways of ECs in the normal and OIR groups showed a significant decrease in the Notch signaling pathway between these two groups (Fig. 5A). Based on our previous work [4, 6, 8], the Notch signal plays an essential regulatory role in angiogenesis and maintaining endothelial homeostasis. Therefore, we tested several molecules in the Notch signaling pathway in the HUVECs stimulated by rhGal3 (Fig. 5B). The results showed that compared with the control group, the downstream molecules of the Notch signal such as Hes1 and Hey1 were significantly downregulated at 6 h after the supplement of rhGal3, indicating Gal3 has a pro-angiogenic effect through the Notch signaling pathway. The Notch receptor, Notch1, was also upregulated at 6 h and its ligand Jag1 was downregulated, which suggested that Gal3 had affected the combination of Notch1 and Jag1. Exogenous rhGal3 was then added to HUVECs and the co-location of rhGal3 and Jag1 expressed by HUVECs could be observed in vitro by IF (Fig. 5C). Further, after constructing a molecular docking prediction model, we noticed the binding sites of Jag1 and Gal3 were very close to the binding region of Jag1 and Notch1 (Fig. 5D), indicating that Gal3 might disturb the combination of this pair of Notch ligand and receptor. Therefore, we conducted immunoprecipitation (IP) to confirm the interaction between these proteins. The results confirmed our hypothesis that Gal3 tended to combine with Jag1 (Fig. 5E) and that the involvement of Gal3 weakened the combination of Jag1 and Notch1 (Fig. 5F). The combination blocked the conduction of the Notch signaling pathway, resulting in reduced expression of Hes1 and Hey1, and may explain how Gal3 interfered with endothelial homeostasis and regulated endothelial metabolism.

### Microglial Gal3 contributed to the Notch inhibition induced angiogenic response

**Fig. 6:**
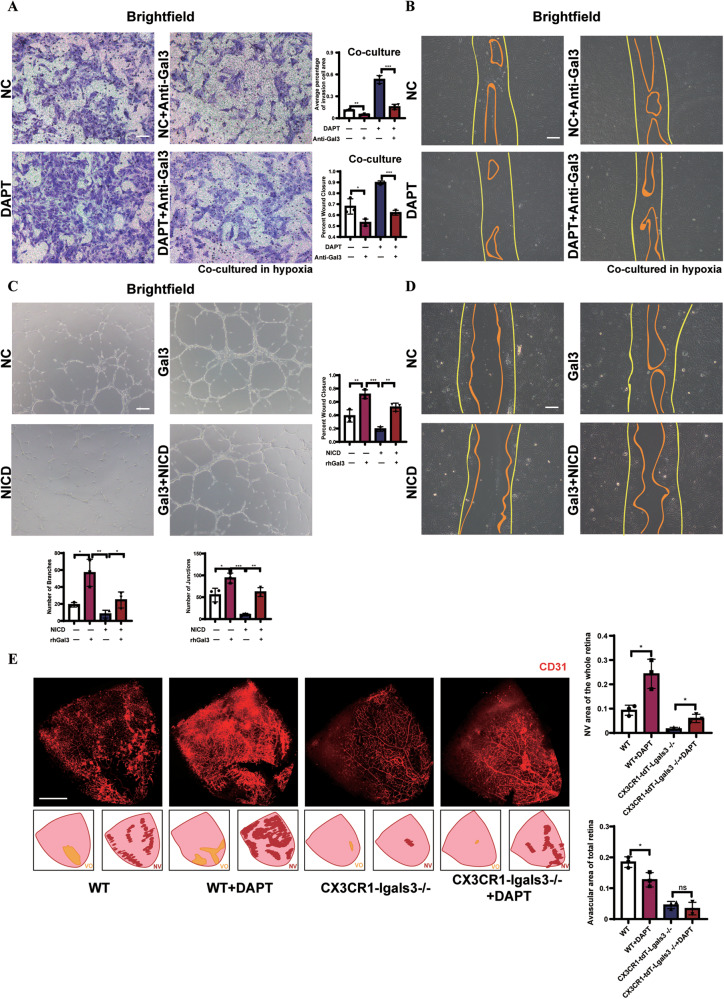
The regulation of gal3 was reversed by Notch interfering. The bEnd.3 cells were precultured with DAPT and DMSO as a control for 24 h and then were cocultured with BV2 in a hypoxic condition with or without anti-Gal3 for 12 h. The capacity of cell migration was evaluated by transwell assay (**A**) and wound closure area after a scratch (**B**). (*n* = 3 per group). Scale bar, 100 µm. HUVECs infected with Ad-NICD and Ad-Ctr were cultured by ECM with or without rhGAl3 48 h after infection. The capacity of lumen formation after 6 h (**C**) and cell migration after 12 h (**D**) was detected. (*n* = 3 per group). Scale bar, 100 µm. **E** The WT and CX3CR1-Lgals3 mice were subjected to DAPT intraperitoneal injection on P12, P14, and P16 when the OIR model was constructed on the mice. Retina samples were collected from the WT and CX3CR1-Lgals3 mice on P17, followed by immunostaining for retinal vessels (red) (*n* = 3 per group). Scale bar, 500 µm. Bars = means ± SD; *P < 0.05; **P < 0.01; ***P < 0.001, NS no significance.

DAPT is an effective inhibitor of γ-secretase which inhibits spontaneous ligand-dependent Notch activation [8]. We cocultured endothelial bEnd.3 cells precultured with DAPT and activated BV2 cells in hypoxia. The capacity for cell invasion and cell migration was significantly enhanced after Notch inhibition by DAPT and was reduced when the Gal3 secreted by BV2 was neutralized by anti-Gal3. In the group treated with both the anti-Gal3 and DAPT, the capacities for cell invasion and cell migration of bEnd.3 cells were enhanced compared with those treated only by DAPT, indicating that the neutralization of Gal3 partially rescued the effect of DAPT-induced Notch inhibition in bEnd.3 cells (Fig. 6A, B). Moreover, when we transfected HUVECs with adenovirus vector-expressing Notch-intracellular domain (Ad-NICD) to activate Notch signal, decreased cell migration and invasion were observed. When Gal3 was added, these effects were partially alleviated, indicating the function of Gal3 in rescuing Notch activation by Ad-NICD in HUVECs (Fig. 6C, D).

To further confirm that Gal3 may inhibit angiogenesis by interfering with Notch signaling, DAPT intraperitoneal injection was used to disrupt the Notch signal in vivo in both WT OIR mice and CX3CR1-lgals3−/− OIR mice (Fig. S3C, D). Notch inhibition aggravated retinal angiogenesis via expanded vessel density and increased neovascular clusters in WT OIR mice (Fig. 6E). However, we observed that pathological angiogenesis was markedly regressed in the retina of CX3CR1-lgals3-/- OIR mice with DAPT injection, indicating that Gal3 could contribute to the Notch inhibition-induced angiogenic response.

### Pathological neovascularization was alleviated after Gal3 inhibition

**Fig. 7:**
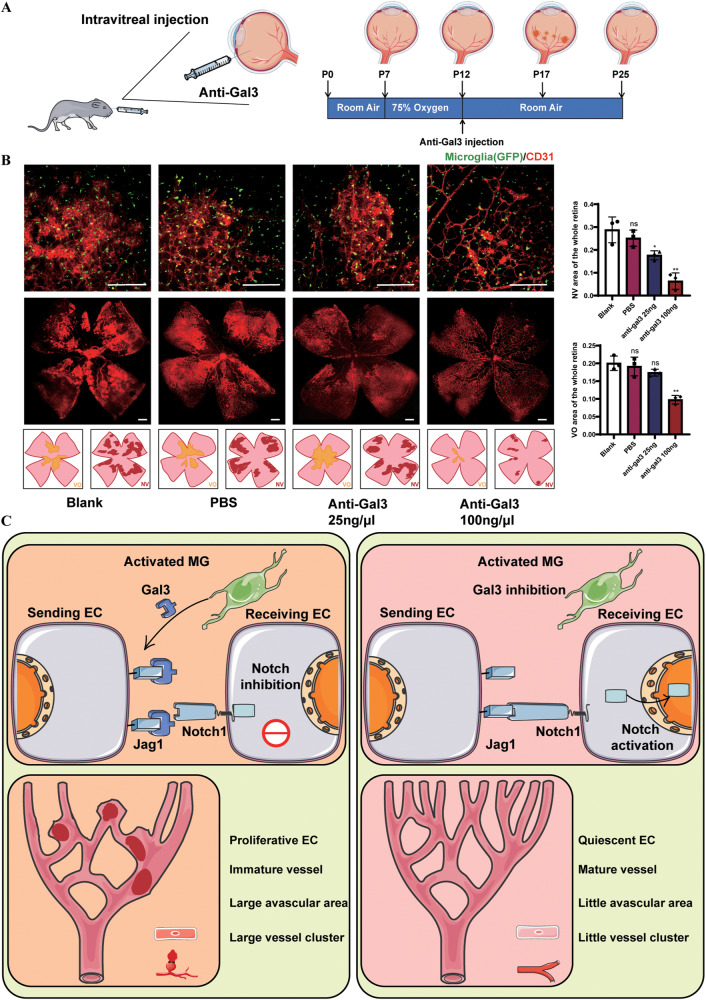
Gal3 inhibition alleviated retinal angiogenesis. **A** CX3CR1-GFP mice were subjected to intravitreous injection of PBS, and anti-Gal3 with 25 and 100 ng/μl on P12 when the OIR model was constructed. **B** IF staining of retinal vessels (CD31, red) and microglia (CX3CR1, GFP) on P17 in the flat-mounted and cross-section from the blank, PBS, and anti-Gal3(25 and 100 ng/μl) group. (*n* = 3 per group). Scale bar, 200 µm. **C** Schematic illustration of the mechanism by which microglia-derived Gal3 promoted retinal angiogenesis. Bars = means ± SD; *P < 0.05; **P < 0.01, NS no significance.

With Gal3 as a potential therapeutic target, we alleviated neovascular diseases by local injection of Gal3 neutralizing antibody in vivo. Phosphate-buffered saline (PBS), and anti-Gal3 were injected into the vitreous of the OIR mice on P12 (Fig. 7A), while a control group received no treatment. No significant difference was found between the PBS and control groups, eliminating bias due to the procedure. After injection of anti-Gal3, the NV area and the VO area decreased significantly, especially at the high concentration of Gal3 (Fig. 7B), indicating a significant effect of Gal3 as a target in the treatment of retinal angiogenesis and the treatment effect was dose-dependent. The inhibition was possible because the Gal3 released by activated microglia competitively combined with Jag1, resulting in inhibition of the Notch1-Jag1 pathway. Thus, the Notch signaling pathway was partly suppressed and its maintenance of vascular homeostasis was impeded. Targeting microglial Gal3 enhanced Jag1-Notch signaling, activating ECs to become quiescent and therefore inhibiting the angiogenic response (Fig. 7C).

## Discussion

This study has uncovered a Gal3-dependent paracrine relationship between microglia and neighboring ECs in pathological retinal angiogenesis. We have shown for the first time an increased expression of exogenous Gal3 from activated microglia in ischemic stress. Gal3 subsequently bound to Notch ligand Jag1, thus eliciting competitive inhibition of Notch signaling in ECs, promoting EC activation and angiogenic metabolic changes. Furthermore, inhibition of microglial Gal3 significantly suppressed the angiogenic response in retinal neovascularization. Our findings provide novel mechanistic insights on microglia in retinal neovascularization and identify Gal3 as a new therapeutic target for the potential treatment of retinal angiogenesis.

In ischemic conditions, microglial cells are highly activated and aggregated at sites of retinal injury, where they exert diverse functions and interact with other retinal cells. For instance, after retinal ischemia/reperfusion injury, IL-4 promotes microglial polarization toward a neuroprotective phenotype [49], whereas the disruption of transforming growth factor-β (TGF-β) signaling in microglia exacerbates retinal neovascularization in OIR by promoting leukocytosis and Igf1 expression [50]. In the current study, we ablated microglia from the OIR retina from P12 via PLX5622, an inhibitor of CSF1R tyrosine kinase. By inhibiting the binding of CSF1 and CSF1R, the development, maintenance, and activation of microglia were inhibited [51]. The removal of microglia selectively inhibited pathological neovascularization in the OIR model, illustrating the pivotal contribution of microglia in OIR. Moreover, CX3CR1 + CCR2- microglia typically expressed high levels of Gal3 in OIR groups[52, 53], a protein known to be a potent immunomodulator in neuroinflammatory disorders [27, 52]. This indicates that microglia-derived Gal3 functioned as an important mediator in the regulation of retinal vascularization in ischemia.

Galectins are a class of evolutionarily conserved proteins, which bind to glycosylated proteins and lipids and can be located extracellularly, in the cell membrane, or intracellularly [28]. Among the galectin family, Gal3 is used as a putative marker for activated brain microglia and macrophages [54]. As well as a marker, Gal3 is upregulated in many central nervous system (CNS) and retinal diseases associated with inflammatory response including Alzheimer’s, stroke, diabetes, and hypoxia/ischemia [55–58]. In microglia-specific Gal3 knock-out mice, we observed that hyperoxia-induced angiogenesis was greatly attenuated in the OIR model, suggesting a pro-angiogenic function of Gal3 in microglia.

Studies showed that Gal3 can dynamically regulate the function of cell surface glycosylation receptors by its long chain, aperiodic, and dynamically changing proline-rich N-terminal tail (NT) [36]. An important role of Gal3 on the cell surface is to crosslink and recombine glycosylated membrane proteins [59], such as binding to insulin receptors, to participate in intercellular signals [60]. Notch ligand activity can also be indirectly regulated by ligand expression signaling or posttranslational modification, serving to spatiotemporally compartmentalize Notch signaling activity and integrate it into a molecular network that orchestrates developmental events [11, 61]. Interestingly, the ligand/receptor of Notch has a variety of glycosylation modifications such as *O*-glucan, *O*-fucoidan, and *N*-glycan [37, 38], providing a glycosylation site for Gal3 to bind. Notch1 and Gal3 regulate the activation of reactive astrocytes after brain injury, and their mechanism depends on the combination of the Notch1 intracellular segment (NICD1) and Gal3 [62]. Similarly, Bernardes et al. [63] revealed that Gal3 regulates the activation of bone marrow-derived dendritic cells and influences the polarization of T helper responses via Notch/Jag1 pathway. As the Notch signaling pathway is essential for the regulation of vascular ECs and ocular neovascular diseases [4, 6, 9], it is probable that microglial Gal3 regulation of angiogenesis is interactive with the Notch signaling pathway. Through IP and immunofluorescent staining, we confirmed that microglia-derived Gal3 bound to Notch ligand Jag1 in ECs, thus blocking Notch signaling and promoting endothelial proliferation and angiogenesis.

Quiescent ECs (QECs) maintain the integrity, tone, and redox homeostasis of the vascular barrier [64]. While an extraordinary change from quiescent to highly migratory and proliferative can be observed during angiogenesis [65]. Recent research has shown that the changes in the metabolic level of ECs might also be the driving force for this phenotypic switch, governed by the Notch signal [66, 67]. The present study showed that Gal3 secreted by microglia regulated the metabolic level of ECs and promoted endothelial proliferation, at least partly by inhibiting the Notch signaling pathway. The simultaneous regulation of Gal3 on endothelial metabolism and cellular phenotypes suggests potential metabolism-phenotype relationships and indicates that ECs may be regulated by adjacent cells. Metabolic cross-talk between ECs and their adjacent cells are a prominent problem for future research, especially in the diseases related to EC dysfunction, such as cancer, atherosclerosis, and ocular neovascular diseases. It also suggests potential targets such as Gal3 for the treatment of vascular diseases besides VEGF inhibition, indicating a direction for future research.

## Materials and methods

### Animals

Lgals3 heterozygous conditional knockout mice (C57BL/6J) and CX3CR1-Cre homozygotes mice (C57BL/6J) were purchased from Cyagen (Suzhou, China). The CX3CR1-GFP mice and CX3CR1-CreERT-tdT mice were maintained in our laboratory. PCR genotyped littermates to obtain CX3CR1-Cre-Lgals3 (control; Ctr) and CX3CR1-CreERT-Lgals3 mice. Mice were injected intraperitoneally with tamoxifen (100 mg/kg; Sigma Aldrich, St. Louis, MO, USA) injection once a day and continuously from P3 to P7 [68].

An oxygen-induced retinopathy (OIR) model was induced as previously described [69]. Mouse pups were exposed to hyperoxia (75% oxygen) from postnatal day 7 (P7) to P12 in order to induce vaso-obliteration and returned to room air. The maximal preretinal NV occurred at P17, followed by a phase of vascular regression almost completed at P25. At P12, 1 μl gal3 antibody (GeneTex Cat# GTX41041, RRID: AB_11163402) was injected intravitreally at the concentration of 25 ng/μl and 100 ng/μl in the right eye of pups with PBS or no intervention in the left as a control. For CSF1R inhibitor PLX5622 (Selleckchem, Houston, TX, USA) treatment, CX3CR1-GFP mice were treated daily by oral gavage with 100 μl solution (10 mg/ml) per 10 g body weight (with the final dose of 100 mg/kg body weight) beginning on P12 when the OIR model was constructed. DAPT intraperitoneal injection was administrated at the dose of 30 mg/kg on P12, P14, and P16. Newborn mice were randomly chosen for the experiments using a table of random numbers. The sample size is estimated to effectively detect a significant difference among the groups. The investigator was blinded to the group allocation.

### Cell isolation, culture, and transfection

HUVEC (RRID: CVCL_9Q53) at passages 3–4 were used and cultured in EC medium (ECM) (ScienCell, California, USA) with 5% FBS, 1% EC growth supplements (ECGS), 100 μg/mL streptomycin, and 100 U/mL penicillin. All the cells were placed in a humidified atmosphere with 5% CO2 at 37 °C. For cytokine stimuli, HUVECs were starved in ECM with 0.5% ECGS and without FBS for 6 hours (6 h) and then treated with rhGal3 (Peproptech, Cranbury, NJ, USA) at 50 ng/mL and were harvested at 0, 6, 12, and 24 h.

The mouse microglia cell lines of BV2 were provided by the Institute of Neurosciences at the Fourth Military Medical University, and were originally obtained from the cell bank of the Chinese Academy of Science (Shanghai, China). The mouse microglia cell lines (BV2 cells) were cultured in Dulbecco’s modified eagle medium (DMEM) containing 10% FBS and 100 μg/mL streptomycin and 100 U/mL penicillin.

Small interfering RNA (siRNA) of mouse Gal3 (siGal3) (GenePharma, Shanghai, China) was selected from three sets for in all the vitro studies described in this manuscript (sense [5’-3’]: CCUUCUUGUAAGACAUCCATT, and antisense [5’-3’]: UGGAUGUCUUACAAGAAGGTT). The siGal3 used in the coculture system was the effective one. Then, 5 nmol negative control (NC) or siGal3 was dissolved in 25 μL RNA-free water to prepare the working solution. BV2 cells were transfected with NC or siGal3 at a concentration of 100 nM by Lipofectamine 2000 reagent (Invitrogen, Carlsbad, CA, USA), following the manufacturer’s instructions. The transfected BV2 cells were cultured in hypoxia.

The bEnd.3 cells were maintained in our laboratory and cultured in Dulbecco’s modified eagle medium (DMEM) containing 10% FBS and 100 μg/mL streptomycin and 100 U/mL penicillin. In the rescue part of interfering the expression of Notch signal and Gal3 at the same time, BV2 was precultured by complete DMEM under hypoxia for 12 h and HUVECs were precultured by complete ECM with DAPT (25 μM) or DMSO for 48 h. The coculture system was placed in hypoxia (5% CO_2_ and 1% O_2_). In addition, Ad-NICD (Hanbio, Shanghai, China) was transfected in HUVECs as described in our previous report [8].

### Lumen formation assay

HUVECs were seeded in 48-well plates (1 × 10^5^ cells/well) precoated with 200 μL Matrigel (1:1, BD Biosciences, Bergen County, NJ, USA) and incubated at 37 °C for 4 h. ImageJ was used to measure the number of branches, loops, and total length of the cell cords.

### Cell invasion assay

Transwell invasion assay was used to observe and test cell invasion ability based on a published protocol [9]. HUVECs were seeded at a total number of 1.0 × 10^4^ in 200 μl ECM containing 0.5% FBS without ECGS in the upper chamber. BV2 was seeded at a total number of 1.0 × 10^6^ with ECM containing 0.5% FBS without ECGS in the lower chamber. Cells were cocultured for 12 h in hypoxia and were fixed by 4% PFA for 10 min and stained with crystal violet for 30 min. A microscope (CKX41, Olympus, Tokyo, Japan) with a CCD camera (DP70, Olympus, Tokyo, Japan) was used to observe the number of HUVECs invading the lower chamber.

### Cell migration assay

Cell migration ability was tested as described previously [70]. Briefly, a p20 pipette tip was used to create a scratch, and the medium was then replaced by ECM supplemented without FBS and with 0.5% ECGS, with or without rhGal3 (or cocultured with MG as explained earlier, or cultured by conditioned media). A microscope (CKX41, Olympus, Tokyo, Japan) with a CCD camera (DP70, Olympus, Tokyo, Japan) was used to observe wound closure and measure the length of the scratch at 0, 6, 12, and 24 h after the scratch was made.

### Cell proliferation determinations

Cell proliferation determination was tested by using Cell Counting Kit-8 (CCK8, Bimake, Houston, TX, USA) according to the manufacturer’s protocol. In short, bEnd.3 cells were cultured in 96-well plates with conditioned medium from BV2 cells transfected with siRNA. After 0, 6, 12, and 24 h in culture, the CCK8 solution was added to each well for 1 h. The optical density (OD) at a wavelength of 450 nm was measured in each well.

### Flow cytometry

Collagenase I at 1 μg/ml was used to digest the retinal collagen in CX3CR1-GFP mice at 37°C for 40 min so that the retinal cells were separated. PBS buffer containing 0.5% fetal bovine serum and 0.1% sodium azide was used to resuspend the whole retinal cells. All the samples were computer analyzed and the 10^3^ specific gate in FACS was used to count the number of GFP+ microglia.

### Plasmid construction

The Gal3 promoter (NM_001357678), Notch1 promoter (NM_017617.5), and Jag1 promoter (NM_000214) were amplified by PCR with HUVECs DNA as a template, and subcloned into the pGL3-basic plasmid (Promega, Madison, WI, USA) to construct pGL-Gal3 and pGL-Jag1. Amplified fragments were cloned into the pGL3-promoter plasmid (Promega, Madison, Wisconsin, USA) to construct a correspondent plasmid. HEK293T cells were co-transfected with pGL-Gal3 and pGL-Jag (100 ng).

### Fluorescence in situ hybridization

10% neutral buffered formalin was used to fix tissues at room temperature for 24 h, and paraffin was used to dehydrate and embed the tissues. Tissue sections were cut at 5-μm thickness and the RNA scope 2.5 HD Assay-RED Kit was used to detect RNA in situ according to the manufacturer’s instructions (ACDBio, Santa Clara Valley, CA, USA) [71]. Sequences of the probes used in the study are as follows: Lgals3 (NM_010705.3, 617-650, 5’-TGACTCTCCTGTTGTTCTCATTGAAGCGGGGGTT-3’).

### Immunostaining

First, 4% paraformaldehyde (PFA) was used to dissect and pre-fix retinas overnight at 4 °C. PBS containing 1% bovine serum albumin (BSA) and 1% Triton X-100 was used to block and permeabilize the samples overnight. We incubated samples with anti-CD31 (BioLegend Cat# 102502, RRID:AB_312909), anti-gal3 (Abcam Cat# 2401-1, RRID:AB_1267156), Iba1 (FUJIFILM Wako Shibayagi Cat# 019-19741, RRID:AB_839504), Jag1 (Santa Cruz Biotechnology Cat# sc-390177, RRID:AB_2892141) in PBS containing 1% BSA and 1% Triton X-100 overnight at 4 °C, followed by incubation with Alexa Fluor®647 goat anti-rabbit immunoglobulin G (IgG; H + L) secondary antibody (1:100) and Alexa Fluor®594 donkey anti-rat immunoglobulin G (IgG; H + L) secondary antibody (1:100) in PBS. DAPI staining was performed according to the manufacturer’s instructions. Between each step, PBS was used to wash the samples three times for 10 min. Retinas were flat-mounted under a dissecting microscope (Olympus) and a confocal laser scanning microscope (FV1000, Olympus) was used to take photographs. Antibody reagents are listed in Supporting Table S2.

### Analysis of energy metabolism

Seahorse Extracellular Flux XFp Analyzer (Agilent Technologies, Palo Alto, CA, USA) was used to measure bioenergetic status. OCR was measured using the XF cell Mito Stress test (Agilent). HUVECs were seeded at the density of 30,000 cells per well in triplicate on 24-well poly-d-lysine-coated plates and cultured with ECM with or without rhGal3 for 24 h at 37 °C in a 5% CO_2_ atmosphere. Unbuffered Base Medium (Agilent) supplemented with 2 mM glutamine (Invitrogen), 2 mM pyruvate (Sigma, St. Louis, Missouri, USA), and 7.1 mM glucose (Sigma) was added after the medium was removed before the experiment. The whole system was adjusted to NaOH at pH 7.4 for 1 h at 37 °C. Basal conditions, sequential injections of oligomycin (1 µM final concentration), FCCP (fluoro 3-carbonyl cyanide-methoxyphenyl hydrazine; 1 and 2 µM final concentrations) and a mix of rotenone and antimycin A (1 µM final concentration) were measured following the manufacturer’s recommendations. The acidification rate of the extracellular environment (ECAR) was measured using the XF Glycolysis stress test (Agilent). ECAR was measured after the addition of glycolysis modulators (10 mM glucose, 1 µM oligomycin, and 100 mM 2-deoxy-glucose).

### scRNA-seq

Single-cell suspensions from disaggregated mice retina were assessed for cell quantity and cell viability by Trypan Blue (YEASEN Biotech Co., Ltd, Shanghai, China). The conduction of Single-cell RNA sequencing was performed by the Chromium (10X Genomics) instrument. The Chromium Single Cell 3′ V3 Library & Gel Bead Kit (10X Genomics) was used to analyze the sample. Gene expression libraries were sequenced using the NoveSeq 6000 (Illumina), conducted by Gene Denovo Biotechnology Co., Ltd (Guangzhou, China). Raw gene expression matrices were combined in R (version 3.5.3) mapping to GRCm38, which was then converted to a Seurat object using the Seurat R package (version 3.0.1). All cells were removed that had either more than 40,000 UMIs, over 8000 or below 200 expressed genes, or over 25% UMIs derived from the mitochondrial genome.

### Quantitative real-time PCR

TRIzol was used to prepare all sections and the PrimeScrip RT reagent kit (TaKaRa, Dalian, China) was used to reverse-transcribed the samples into cDNA followed by the instruction. We used the SYBR premix ExTaqII (TaKaRa) and Applied Biosystems 7500 Real-time PCR system (Applied Biosystems, Carlsbad, CA, USA) to perform quantitative real-time PCR (qPCR). We chose β-actin as a reference control. Primers are listed in Supporting Table S1.

### Western blotting

We used the radioimmunoprecipitation assay lysis buffer to extract total protein, which contained 10 mM of phenylmethanesulfonyl fluoride. The cell supernatant was concentrated 10 times by ultrafiltration tube (Millipore, MA, USA). A bicinchoninic acid assay protein assay kit (Thermo Fisher Scientific, Waltham, MA, USA) was used to quantify total protein in cells and retinal tissues. We used Sodium dodecyl sulfate/polyacrylamide gel electrophoresis to separate the samples and then the samples were transferred onto polyvinylidene fluoride membranes. Then we incubated the blottings with primary antibodies at 4°C overnight followed by HRP-conjugated secondary antibodies at 37 °C for 2 h. We developed the blottings with enhanced chemiluminescence (Clinx Science Instruments, Shanghai, China). Antibody reagents are listed in Supporting Table S2.

### Immunoprecipitation

We performed immunoprecipitation as described previously [72]. In brief, total cell lysates were collected and then were immunoprecipitated by incubation with antibodies against Flag (1:100, Cell Signaling Technology, Boston, MA, USA) and HA (1:100, Cell Signaling Technology). SDS–PAGE was used to separate immunoprecipitates and then was blotted onto a polyvinylidene difluoride (BioRad Laboratories, Hercules, CA, USA) membrane. The membrane was incubated with antibodies against HA (1:2000, Cell Signaling Technology) and Flag (1:2000, Cell Signaling Technology) respectively and visualized by enhanced chemiluminescence (ECL Kit; Amersham Biosciences, Slough, Buckinghamshire, UK). Whole-cell lysates were separated by SDS–PAGE and blotted on polyvinylidene difluoride membranes (Millipore) for western blotting analysis reprobed with appropriate peroxidase-conjugated HA and FLAG antibodies. Blots were visualized by enhanced chemiluminescence (ECL Kit; Amersham Biosciences).

### Molecular docking

MOE 2019 was used to construct the molecular docking prediction model. The sequences were found in the PDB database.

### OIR lesion assessment

OIR lesion was evaluated according to the proposed protocol [73]. Briefly, the NV, VO, and total areas were manually outlined following the protocol, and quantified by a masked observer, and the ratio of NV and VO area to total retinal area was then calculated.

### Microglial skeletal analysis

The skeletal analysis plugin was used to analyze microglial skeletal as described previously [74]. Briefly, graphs were pre-processed by converting to 8-bit and applying the FFT bandpass filter in ImageJ. We adjusted the brightness/contrast of the graphs to best visualize the branches of the microglia. We then applied an unsharp mask to further increase the contrast of the photomicrograph and applied the despeckle function to remove noise. We adjusted the threshold and despeckle, close, and applied the remove-outliers functions, then skeletonized the binarized image. Microglial cell somas were counted manually to obtain a total microglial count per photomicrograph. The total number of microglial branches were calculated across the entire image.

### Statistics

Image-Pro Plus 6.0 and GraphPad Prism 5 software were used to perform statistical analysis. All the quantitative data are presented as mean ± SD. Statistical significance was calculated using Student’s *t*-test. The statistical tests above were justified as appropriate. The data were normally distributed with the variation marked in each figure, and the variance was similar between the groups which were statistically compared. The control group was set as 1 when compared in fold values. *P* < 0.05 was considered significant.

## Supplementary information

Table S1

Table S2

Supplementary Figure1

Supplementary Figure2

Supplementary Figure3

Supplementary Figure4

Supplementary Figure5

Supplemental Figure legend

reproducibility checklist

## Data Availability

We have deposited the scRNA-seq data in the NCBI SRA database (BioProject: PRJNA864092) All the data sets and details in this study are available from the corresponding author upon reasonable request.
