# Supplementary material for: Microglial Galectin3 enhances endothelial metabolism and promotes pathological angiogenesis via Notch inhibition by competitively binding to Jag1
Source: Cell Death Dis. 2023 Jun 28;14(6):380. doi: 10.1038/s41419-023-05897-8 (PMC10300109; doi:10.1038/s41419-023-05897-8)
Supplement: Supplementary file 1 — Table S1 [file 41419_2023_5897_MOESM1_ESM.docx]

**Table1. Antibodies used in this study**

| **Name** | **Supplier** | **Clone#** | **Purpose** |
| --- | --- | --- | --- |
| Gal3 | Abcam Cat#2401-1, RRID:AB_1267156 | monoclonal | FACS/IF/WB |
| Ki67 | Abcam Cat# ab92353, RRID:AB_2049848 | monoclonal | IF |
| CD31 | BioLegend Cat# 102502, RRID:AB_312909 | monoclonal | IF |
| Jag1 | Santa Cruz Biotechnology Cat# sc-390177, RRID:AB_2892141 | monoclonal | IF |
| Donkey anti-rabbit Alexa fluo647 | Invitrogen | IgG | IF |
| Donkey anti-goat Alexa fluo594 | LifeTechnologies | IgG | IF/FACS |
| IBA1 | FUJIFILM Wako Shibayagi Cat# 019-19741, RRID:AB_839504 | IgG | IF |
| Anti-Gal3 | GeneTex Cat# GTX41041, RRID:AB_11163402 | IgG | Neutralization |
