# Supplementary material for: Microglial Galectin3 enhances endothelial metabolism and promotes pathological angiogenesis via Notch inhibition by competitively binding to Jag1
Source: Cell Death Dis. 2023 Jun 28;14(6):380. doi: 10.1038/s41419-023-05897-8 (PMC10300109; doi:10.1038/s41419-023-05897-8)
Supplement: Supplementary file 2 — Table S2 [file 41419_2023_5897_MOESM2_ESM.docx]

**Table2. Primers used for qPCR**

| **qPCR** | **Forward(5’-3’)** | **Reverse(5’-3’)** |
| --- | --- | --- |
| Lgals3 | AACACGAAGCAGGACAATAACTGG | GCAGTAGGTGAGCATCGTTGAC |
| CX3CR1 | TAAGACCACAGGCTACGACA | TGAGGTGTGTGATCTTGCATG |
| IL-1β | CGGGAAGACAATAACTGCACCC | CGGTTAGCAGTATGTTGTCCAGC |
| IL-6 | TACCACTTCACAAGTCGGAGGC | CTGCAAGTGCATCATCGTTGTTC |
| GPI | CCATCAAGGTGGACGGCAAAGA | CCGTGATGGATTTGCCAGTGTAC |
| PGK1 | GATGCTTTCCGAGCCTCACTGT | ACCAGCCTTCTGTGGCAGATTC |
| ALDOA | CACGAGACACTGTACCAGAAGG | TTGTCTCGCCATTGGTTCCTGC |
| PKM2 | CAGAGAAGGTCTTCCTGGCTCA | GCCACATCACTGCCTTCAGCAC |
| HES1 | GGAAATGACAGTGAAGCACCTCC | GAAGCGGGTCACCTCGTTCATG |
| HEY1 | TGTCTGAGCTGAGAAGGCTGGT | TTCAGGTGATCCACGGTCATCTG |
| Notch1 | GGTGAACTGCTCTGAGGAGATC | GGATTGCAGTCGTCCACGTTGA |
| Jagged1 | TGCTACAACCGTGCCAGTGACT | TCAGGTGTGTCGTTGGAAGCCA |
| Dll4 | CTGCGAGAAGAAAGTGGACAGG | ACAGTCGCTGACGTGGAGTTCA |
| MSR1 | GCTGTGTCAAGTTTGCCTACCC | CCAGAATGCCATCCAAGGTCTC |
| Clecn4 | CTGCTTCAGTGAAGGGACTATGG | CACTGGTGCTCCAGAAGTTCTC |
| CFP | CGCACGTTCAATGACAGCATCC | GCAAACACAAGGAGGTAGAGAGC |
| β-actin | GGCTGTATTCCCCTCCATCG | CCAGTTGGTAACAATGCCATG |
