# Supplementary figures and images for: Microglial Galectin3 enhances endothelial metabolism and promotes pathological angiogenesis via Notch inhibition by competitively binding to Jag1
Source: Cell Death Dis. 2023 Jun 28;14(6):380. doi: 10.1038/s41419-023-05897-8 (PMC10300109; doi:10.1038/s41419-023-05897-8)

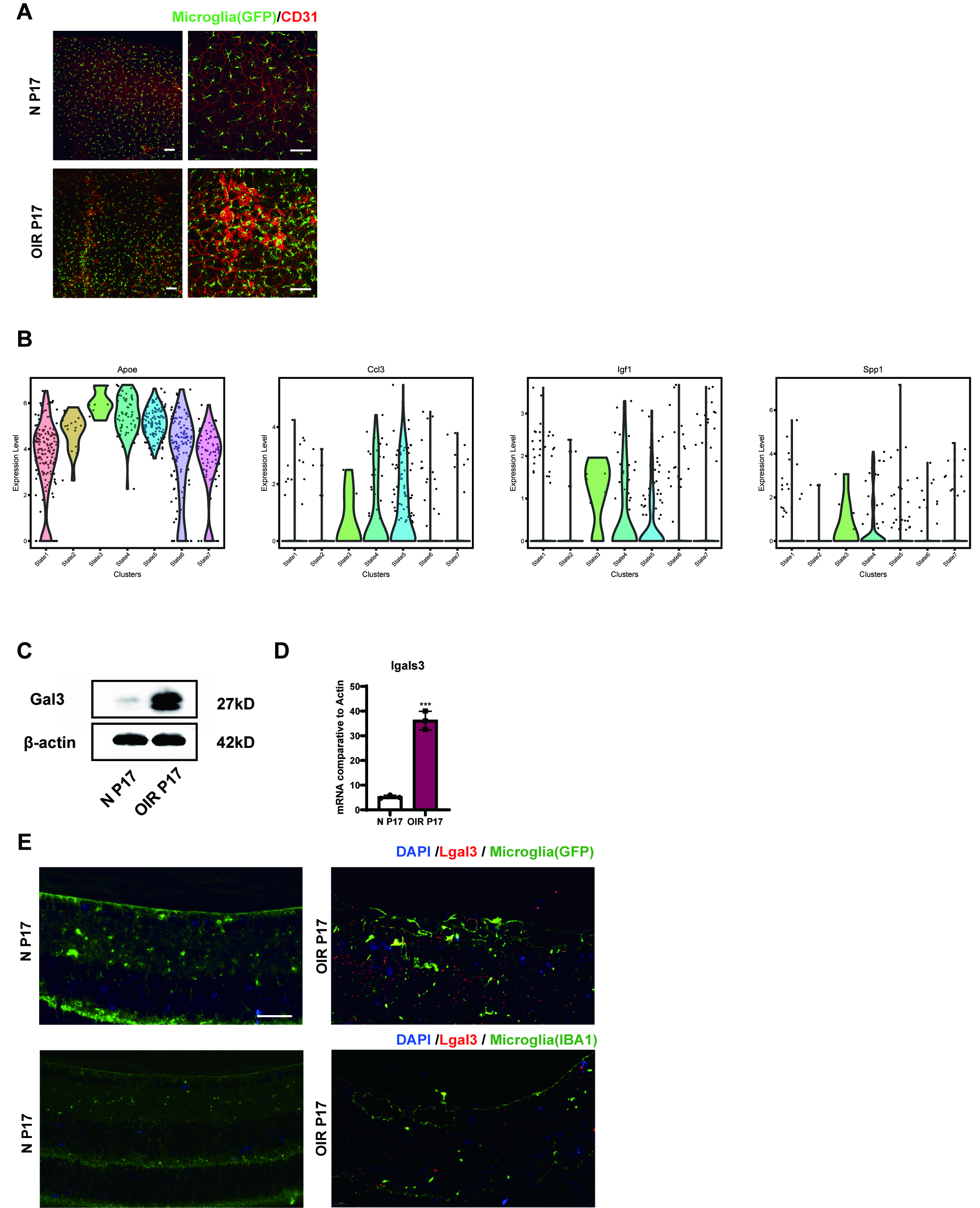

Supplement: Supplementary file 3 — Supplementary Figure1 [file 41419_2023_5897_MOESM3_ESM.tif]

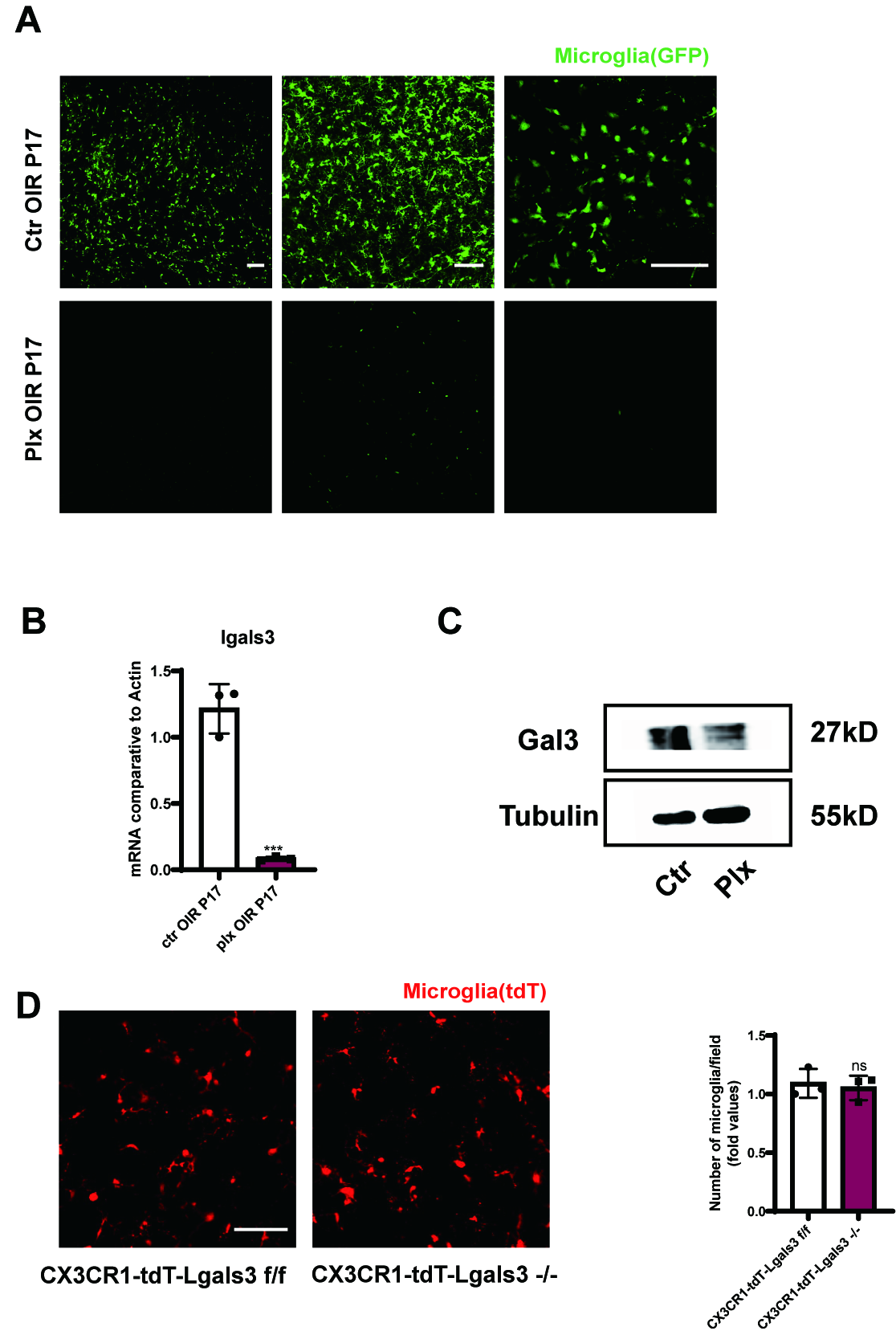

Supplement: Supplementary file 4 — Supplementary Figure2 [file 41419_2023_5897_MOESM4_ESM.tif]

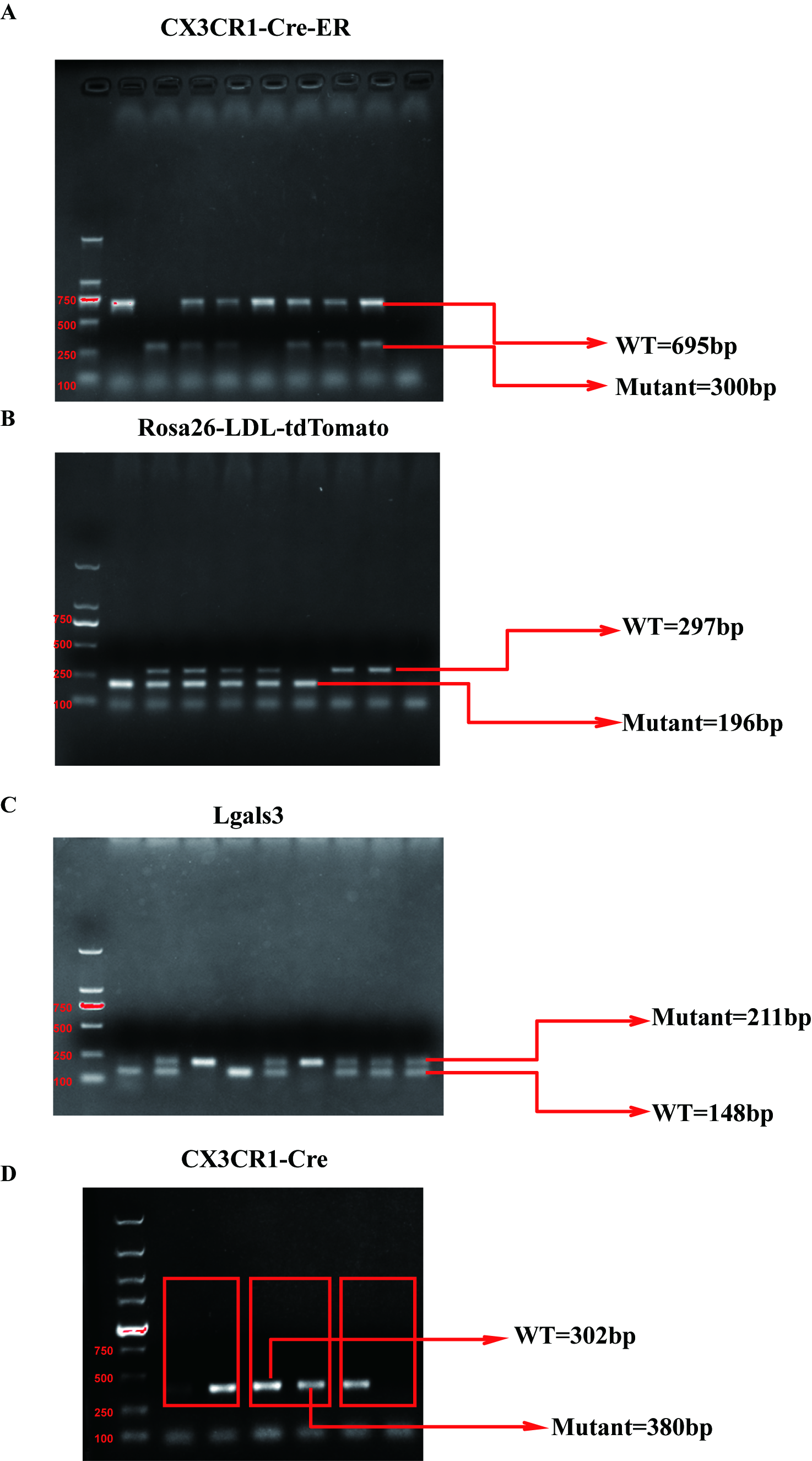

Supplement: Supplementary file 5 — Supplementary Figure3 [file 41419_2023_5897_MOESM5_ESM.tif]

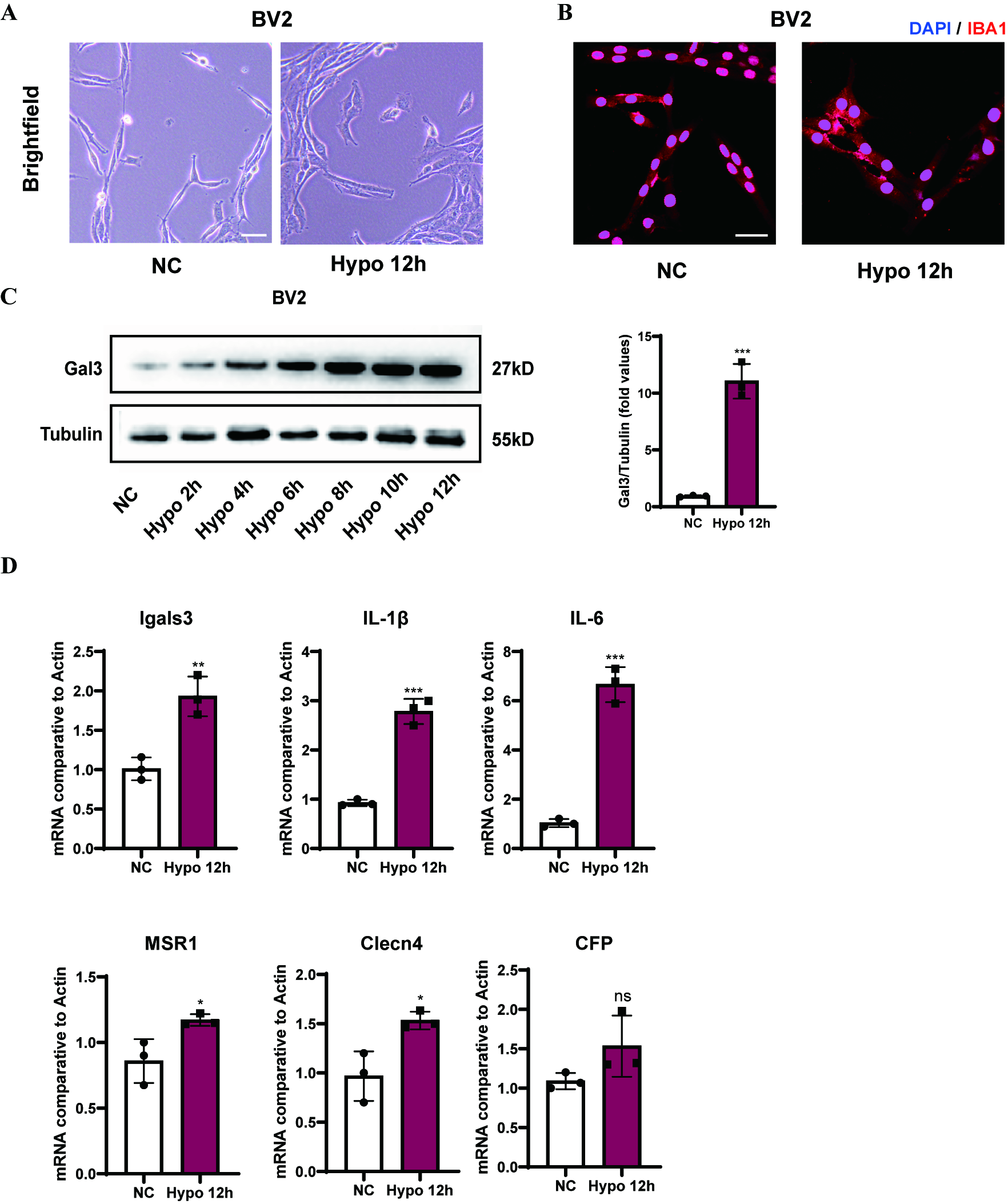

Supplement: Supplementary file 6 — Supplementary Figure4 [file 41419_2023_5897_MOESM6_ESM.tif]

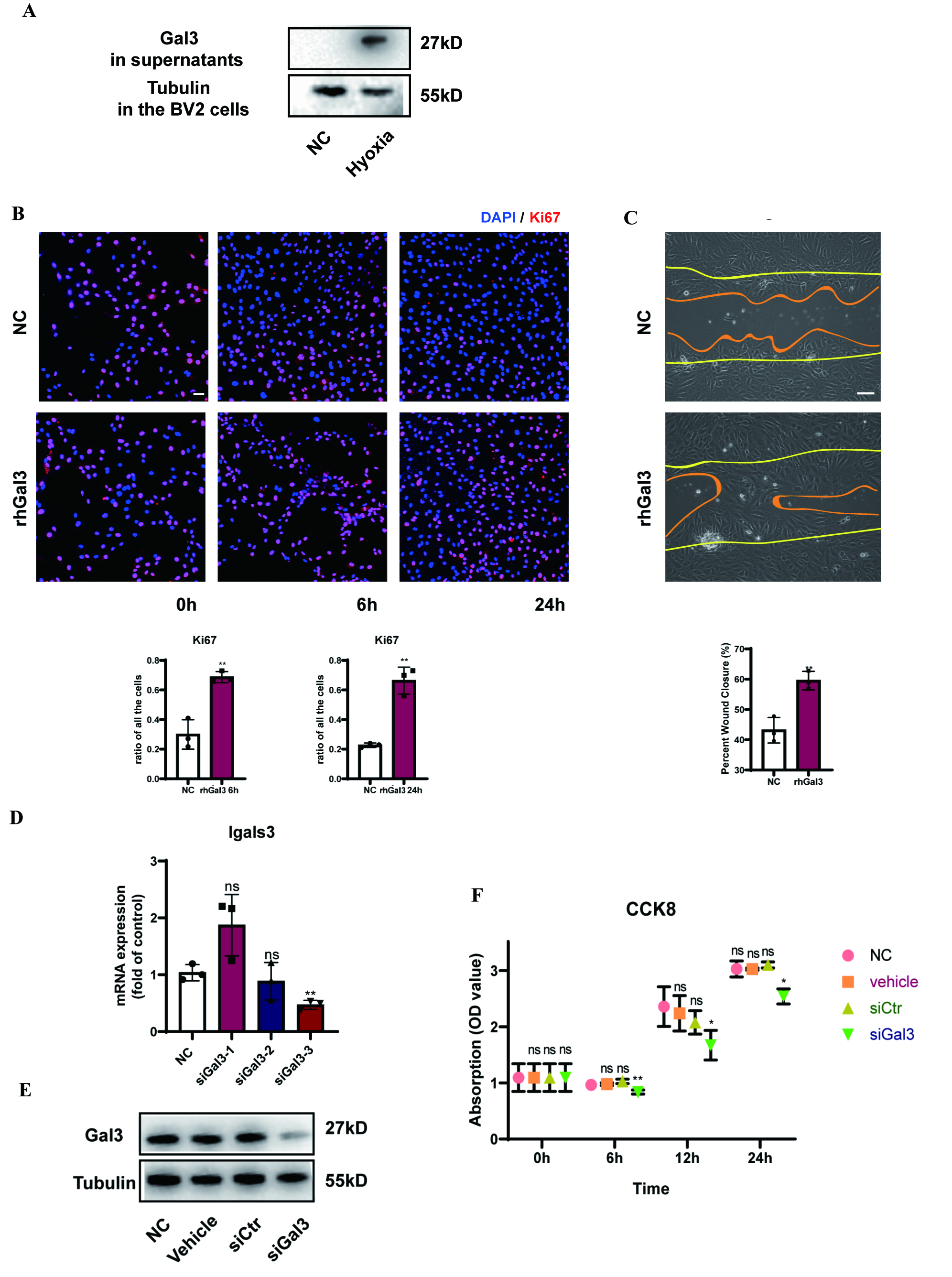

Supplement: Supplementary file 7 — Supplementary Figure5 [file 41419_2023_5897_MOESM7_ESM.tif]
