# Supplementary material for: Microglial Galectin3 enhances endothelial metabolism and promotes pathological angiogenesis via Notch inhibition by competitively binding to Jag1
Source: Cell Death Dis. 2023 Jun 28;14(6):380. doi: 10.1038/s41419-023-05897-8 (PMC10300109; doi:10.1038/s41419-023-05897-8)
Supplement: Supplementary file 8 — Supplemental Figure legend [file 41419_2023_5897_MOESM8_ESM.docx]

**Fig. S1**

(A) Representative micrographs taken by confocal of retinal flat mounts on P17 with immunofluorescence for the morphology of microglia (CX3CR1, GFP) and retinal vessels (CD31, red) on P17 in the normal and OIR group. Scale bar, 100µm. (n=3 per group). (B) Violin plot represented the expression level and frequency among cell clusters for activation-related genes (Apoe, Ccl3, Igf1 and Spp1) in each of the seven states identified by the trajectory analysis in the pesudotime. (C) and (D) Increased expression of Gal3 evaluated by western blotting and qRT-PCR from the whole retinal tissue on P17 in the normal and OIR group. (n=3 per group) (E) FISH of Gal3 assay (red) and immunofluorescent stain of the retinal cross section for nuclei (blue) and microglia (CX3CR1, GFP) in the CX3CR1-GFP mice and the co-staining of microglia with IBA1 (green) and Gal3 assay (red) in the wildtype (WT) on P17 in both normal and OIR groups. Scale bar, 100µm. (n=3 per group). Bars = means ± SD; ***P < 0.001.

**Fig. S2**

(A) Representative micrographs taken by confocal of retinal flat mounts on P17 with immunofluorescence for the morphology of microglia (CX3CR1, GFP) on P17 in both Ctr and Plx OIR groups. (n=3 per group). Scale bar, 100µm. (B) and (C) The expression of Gal3 downgraded after the removal of microglia, determined by qRT-PCR and western blotting on P17 in Ctr and Plx OIR group. (n=3 per group). (D) Representative micrographs taken by confocal of retinal flat mounts on P17 with immunofluorescence for the number of microglia (CX3CR1, tdT) in the WT mice and CX3CR1-tdT-Lgals3-/- mice. (n=3 per group). Scale bar, 100µm. Bars = means ± SD; ***P < 0.001, NS, no significance.

**Fig. S3**

Identification of the genotype of CX3CR1-Cre-ER mice (A), Rosa26-LDL-tdTomato mice (B), Lgals3 mice (C), and CX3CR1-Cre mice (D).

**Fig. S4**

(A)BV2 cultured in DMEM in normoxic and hypoxic conditions. (n=3 per group). Scale bar, 100µm. (B) Immunofluorescence staining for BV2 microglia (IBA1, purple) after 12h induced in hypoxia. (n=5 per group). Scale bar, 100µm. (C) The expression of Gal3 in BV2 microglia elevated after cultured in hypoxia, detected by western blotting every 2 hours (n=3 per group). (D) mRNA expression of lgals3, inflammation-related genes (IL-1β and IL-6) and first-ranking genes (MSR1, Clecn4, and CFP) which were highly expressed in the OIR group in scRNA-seq in vivo (including MSR1, Clecn4, GSN, CFP, and CCR1) were determined in BV2 in normxia and hypoxia by qRT-PCR (n=3 per group). Bars = means ± SD; *P < 0.05; **P < 0.01; ***P < 0.001, NS, no significance.

**Fig. S5**

(A)The content of Gal3 in the conditioned media from the control and hypoxic BV2 cells was determined by western blotting after being concentrated for 10 times. (n=3 per group). (B) IF staining of Ki67 (purple) in HUVECs cultured by normal ECM and ECM with rhGal3. (n=3 per group). Scale bar, 100µm. (C) HUVECs cultured by normal ECM and ECM with Gal3 were wounded with a p20 pipette tip. Photographs were taken immediately and after 8 h (n=3 per group for wound assay and 2 fields were calculated per sample). Scale bar, 100µm. (D) mRNA expression of lgals3 was determined in BV2 microglia in hypoxia after 24h being transfected with a set of siRNA-Gal3 by qRT-PCR (n=3 per group). (E) The expression of Gal3 in BV2 microglia in hypoxia after 48h being transfected with siRNA-Gal3-3, siCtr, and vehicle(lipo2000) was detected by western blotting. (n=3 per group). (F) CCK8 kit was used to determine the proliferation of bEnd.3 cells cultured in conditioned media of BV2 microglia cultured in hypoxia after 48h being transfected with siRNA-Gal3, siCtr, and vehicle(lipo2000). (n=3 per group). Bars = means ± SD; *P < 0.05; **P < 0.01; NS, no significance.
